# Self-Regulated Bilateral Anchoring Enables Efficient Charge Transport Pathways for High-Performance Rigid and Flexible Perovskite Solar Cells

**DOI:** 10.1007/s40820-025-01846-6

**Published:** 2025-07-14

**Authors:** Haiying Zheng, Guozhen Liu, Xinhe Dong, Feifan Chen, Chao Wang, Hongbo Yu, Zhihua Zhang, Xu Pan

**Affiliations:** 1 School of Materials Science and Engineering, Dalian Jiaotong University, Dalian, 116028 People’s Republic of China; 2 State Key Laboratory of Fine Chemicals, School of Chemistry, Dalian University of Technology, Dalian, 116024 People’s Republic of China; 3 Key Laboratory of Photovoltaic and Energy Conservation Materials, Institute of Solid State Physics, Hefei Institutes of Physical Science, Chinese Academy of Sciences, Hefei, 230031 People’s Republic of China; 4 Institutes of Physical Science and Information Technology, Anhui University, Hefei, 230601 People’s Republic of China

**Keywords:** Perovskite solar cells, Buried interface, Bilateral bonding, Defects passivation, Electron transport pathway

## Abstract

Interface modification has been demonstrated as an effective means to enhance the performance of perovskite solar cells. However, the effect depends on the anchoring mode and strength of the interfacial molecules, which determines whether long-term robust interface for carrier viaduct can be achieved under operational light illumination. Herein, we select squaric acid (SA) as the interfacial molecule between the perovskite and SnO_2_ layer and propose a self-regulated bilateral anchoring strategy. The unique four-membered ring conjugated structure and dicarboxylic acid groups facilitate stable hydrogen bonds and coordination bonds at both SnO_2_/SA and SA/PbI_2_ interfaces. The self-transforming property of SA enables the dynamic bilateral anchoring at the buried interface, ultimately releasing residual stress and constructing a stable interfacial molecular bridge. The results show that SA molecular bridge not only can effectively inhibit the generation of diverse charged defects but also serves as an effective electron transport pathway, resulting in improved power conversion efficiency (PCE) from 23.19 to 25.50% and excellent stability at the maximum power point. Additionally, the PCEs of the flexible and large-area (1 cm^2^) devices were increased to 24.92% and 24.01%, respectively, demonstrating the universal applicability of the bilateral anchoring to PSCs based on different substrates and larger area.

**Supplementary Information:**

The online version contains supplementary material available at 10.1007/s40820-025-01846-6.

## Highlights

- Robust interface molecular bridge was constructed by employing self-transforming squaric acid (SA) to reduce residual stress and passivate defects at the buried interface.
- Attributing to the efficient charge transport pathways, the SA-modified perovskite solar cells demonstrate high photovoltaic performance with power conversion efficiency up to 25.50% (rigid) and 24.92% (flexible).
- The SA-modified devices demonstrate excellent stability under various environmental stress conditions, including humidity, thermal aging, light irradiation, and bending.

**Supplementary Information:**

The online version contains supplementary material available at 10.1007/s40820-025-01846-6.

## Graphical abstract

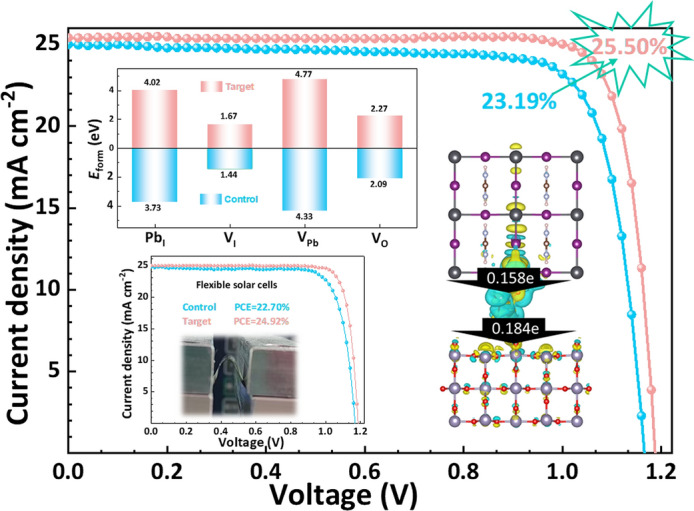

## Introduction

Perovskite solar cells (PSCs) have emerged as highly promising candidates for next-generation photovoltaic technology, characterized by their superior power conversion efficiency (PCE) and cost-effective manufacturing [1–5]. Within PSCs, the buried interface between the electron transport layer (ETL) and the perovskite absorber plays a pivotal role in charge extraction and overall device performance. SnO_2_ has garnered significant attention as a widely employed ETL in PSCs, owing to its exceptional electron transport properties and commendable stability [6–8]. However, despite its advantages, the SnO_2_ is inherently limited by challenges such as a high defect density, diminished charge extraction efficiency, and inadequate coordination [9–11]. These limitations can lead to detrimental outcomes, including charge recombination and a consequential reduction in open-circuit voltage (*V*_oc_), thereby impairing device performance. Furthermore, the buried interface between the perovskite and SnO_2_ substrate critically influences the crystallization kinetics of the perovskite film, which is a factor of paramount importance in optimizing device performance [12–14].

To address these challenges, substantial research efforts have been dedicated to modifying the SnO_2_ buried interface in PSCs through various approaches, including additive engineering, surface modification techniques, and the incorporation of functional groups or nanoparticles [15–18]. For instance, Tian et al*.* introduced a cage polyamine molecule, 1,4-diazabicyclo[2,2,2]octane (DABCO), as a modulator at the SnO_2_ buried interface to regulate the interface properties and enhance device performance [19]. Yang et al*.* developed DL-Carnitine hydrochloride (DL) as a multifunctional interfacial modifier, which effectively passivated buried defects and improved the quality of the perovskite film [20]. Guo et al*.* employed glycocyamine (GDA) as a molecular modifier to establish a molecular bridge at the SnO_2_/perovskite buried interface, resulting in enhanced interfacial performance [21]. These studies collectively aim to achieve better energy level alignment, reduce defect density, and improve charge extraction and device stability. Zhou et al*.* revealed that the performance of perovskite at the buried interface differs significantly from that at the surface and PSCs are primarily illuminated from the bottom during operation, the buried interface exerts a much stronger influence on material properties than the surface [22]. However, due to its non-exposed nature, existing characterization techniques struggle to achieve nondestructive probing of the buried interface [23, 24]. Moreover, Ji et al*.* pointed out that materials commonly employed to modify the buried interface, such as NaI, NaCl, KI and choline iodide (ChI), are susceptible to dissolution or erosion in the dimethylformamide (DMF) and dimethyl sulfoxide (DMSO) solvents of the perovskite precursor solution, which significantly compromises their effectiveness in regulating the buried interface and further increases the challenges associated with studying this critical region [25, 26]. Therefore, the development of a multifunctional buried interface modification material that can remain stable in complex chemical environments and the elucidation of its modulation capabilities are essential for advancing the optoelectronic performance and stability of PSCs.

In this study, we developed a simple yet effective strategy to precisely regulate the SnO_2_-based buried interface by introducing squaric acid (SA) as an interfacial molecular bridge. SA with self-transforming nature can form hydrogen bonds and coordination bonds with both the perovskite and SnO_2_ at the buried interface under different chemical environments, thereby achieving multiple objectives: it passivates charged defects within the perovskite, fills oxygen vacancies (V_O_) on the SnO_2_ surface, and regulates perovskite crystal growth, ultimately yielding high-quality perovskite films with large grain sizes. In addition, SA adjusts the energy levels, serving as an efficient electron transport pathway that facilitates electron transfer from the perovskite to SnO_2_ and reduces carrier recombination. The results demonstrate that SA-treated PSCs exhibit a significantly enhanced power conversion efficiency (PCE) of 25.50%, up from 23.19%, accompanied by improved environmental, thermal, and operational stability. Furthermore, large-area rigid (1 cm^2^) and flexible PSCs based on the multifunctional buried interface achieve high PCEs of 24.01% and 24.92%, respectively. Notably, the bending stability of flexible PSCs is significantly improved due to the release of internal stress, highlighting the practical potential of this approach.

## Experimental Section

### Materials

Lead iodide (PbI_2_) was bought from TCI. Tin (IV) oxide (SnO_2_) colloidal dispersion (15 wt% in H_2_O) was purchased from Alfa Aesar. Formamidine iodide (FAI), methylamine hydrochloride (MACl), methylammonium bromide (MABr), Spiro-OMeTAD, Lithium bis (trifluoromethyl sulfonyl) imine (Li-TFSI) and 4-tert-butylpyridine (tBP) were acquired from Xi’an Polymer Light Technology Corp. Squaric acid (≥ 98%), isopropanol (IPA), lead bromide (PbBr_2_), dimethyl sulfoxide (DMSO), chlorobenzene (CB), dimethylformamide (DMF) and acetonitrile (ACN) were purchased from Aladdin. ITO-PEN with a thickness of about 125 μm was purchased from Peccell Technologies, Inc. (Japan). All materials were used as purchased, without additional purification or modification.

### Device Fabrication

ITO-coated glass substrates were ultrasonically cleaned sequentially in detergent, deionized water, and ethanol (20 min each), followed by oven drying. UV-ozone treatment (20 min) was performed prior to SnO_2_ deposition. A SnO_2_ colloidal dispersion (100 μL of 1 mg mL^−1^ NH_4_Cl in deionized water) was spin-coated (4000 r min^−1^, 30 s), and the films were annealed at 90 °C for 1 h. After that, SA solutions (0, 3, 5, 7 and 9 mg mL^−1^ in deionized water) were spin-coated (4000 r min^−1^, 30 s) onto the SnO_2_ layer, followed by annealing (100 °C, 10 min). After UV-ozone exposure (20 min), the substrates were transferred to a glovebox (RH: 20$–30%) for perovskite deposition. The precursor solution (CsPbI_3_)_0.025_(FAPbI_3_)_0.825_(MAPbBr_3_)_0.15_ (1.5 M Pb^2+^, with 30% MACl) dissolved in a DMF:DMSO (4:1 v/v) mixture was stirred at 60 °C for 2 h. The precursor solution (50 μL) was spin-coated (1100 r min^−1^ for 10 s, 4500 r min^−1^ for 36 s) onto ITO/SnO_2_/SA substrates, with chlorobenzene antisolvent added at the last 15 s. The films were annealed at 105 °C for 50 min. Afterward, the perovskite films were treated with 2-methylthio-2-imidazoline hydroiodide (4 mg mL^−1^ in IPA, 3000 r min^−1^, 20 s) and annealed (100 °C, 10 min) to passivate defects and enhance *V*_oc_ [27]. After cooling to room temperature, Spiro-OMeTAD solution, consists of 73.5 mg spiro-OMeTAD, 29 μL tBP, 17 μL Li-TFSI (500 mg mL^−1^ in ACN) and 8 μL Co^3+^ salt (400 mg mL^−1^ in ACN) in 1 mL CB, was spin-coated (3000 r min^−1^, 30 s). Finally, a 60-nm Au electrode was thermally evaporated (Quorum Q150TE Plus) through a mask (active area: 0.049 cm^2^).

### Characterizations

X-ray photoelectron spectroscopy (XPS) measurements were performed using an ESCALAB Xi + spectrometer (Thermo Fisher Scientific) equipped with an Al Kα X-ray. Ultraviolet photoelectron spectroscopy (UPS) analysis was conducted on an AXIS ULTRA DLD system (Shimadzu/Kratos) with a He I (21.22 eV) excitation source. In-situ GIWAXS data at the spin-coating stage were collected at beamline BL14B1 of the Shanghai Synchrotron Radiation Facility using an X-ray wavelength of 1.54 Å. Fourier transform infrared (FTIR) spectroscopy was carried out with a VERTEX 70v vacuum FTIR spectrometer, while UV–Vis absorption spectra were recorded on a Hitachi U-3900H spectrophotometer. X-ray diffraction (XRD) patterns were obtained by in situ charge and discharge X-ray diffractometer (D2 PHASER). Morphological characterization and cross-sectional analysis of perovskite films and devices were performed by field-emission scanning electron microscopy (FE-SEM, Gemini SEM300). Steady-state photoluminescence (PL) spectra were measured using Fluorescence spectrometer (FLS-1000). The time-resolved photoluminescence (TRPL) spectra were collected by FLS980 with a 485 nm pulsed laser. Grazing Incidence X-ray Diffraction (GIXRD) patterns were acquired by using a Rigaku Smartlab with Cu Kα radiation in the 2*θ* range of 30°-33°. Current density–voltage (*J-V*) curves were measured by a Keithley 2400 digital sourcemeter under AM 1.5G solar light (100 mW cm^−2^), which was provided by an AAA class solar simulator (Newport Oriel Class 3A Model: 94043A). The external quantum efficiency (EQE) spectra were obtained by the quantum efficiency measurement system (QE-R, Enlitech, Taiwan of China). Electrochemical impedance spectroscopy (EIS) measurements (1 Hz-1 MHz) were performed using a Zennium CIMPS-pro setup (Germany). Contact angle measurements were conducted using an OCA15EC goniometer (Dataphysics, Germany). The device's performance was measured using the Candlelight System (Switzerland) under continuous white-LED illumination (1 sun), *J-V* curves were measured every 1 h. Mechanical stability of the flexible devices was tested by a mechanical tester (PR-BDM-100, Puri, China).

### Computational Details

First-principles calculations were conducted using density functional theory (DFT) within the Vienna Ab Initio Simulation Package (VASP) [28, 29]. The exchange–correlation interactions were described by the generalized gradient approximation (GGA) in the Perdew–Burke–Ernzerhof (PBE) formulation [29, 30]. A plane-wave energy cutoff of 480 eV was employed, with a convergence threshold of 10⁻^4^ eV for electronic relaxation. To mitigate spurious interlayer coupling, a 20 Å vacuum layer was introduced. Brillouin zone sampling was performed using a 2 × 2 × 1 Monkhorst–Pack k-point grid. Structural optimization was continued until atomic forces fell below 0.05 eV Å^−1^. Post-processing and structural visualization were facilitated by the VASPKIT and VESTA software packages [31, 32].

## Results and Discussion

### Chemical Interaction and Defect Passivation

**Fig. 1:**
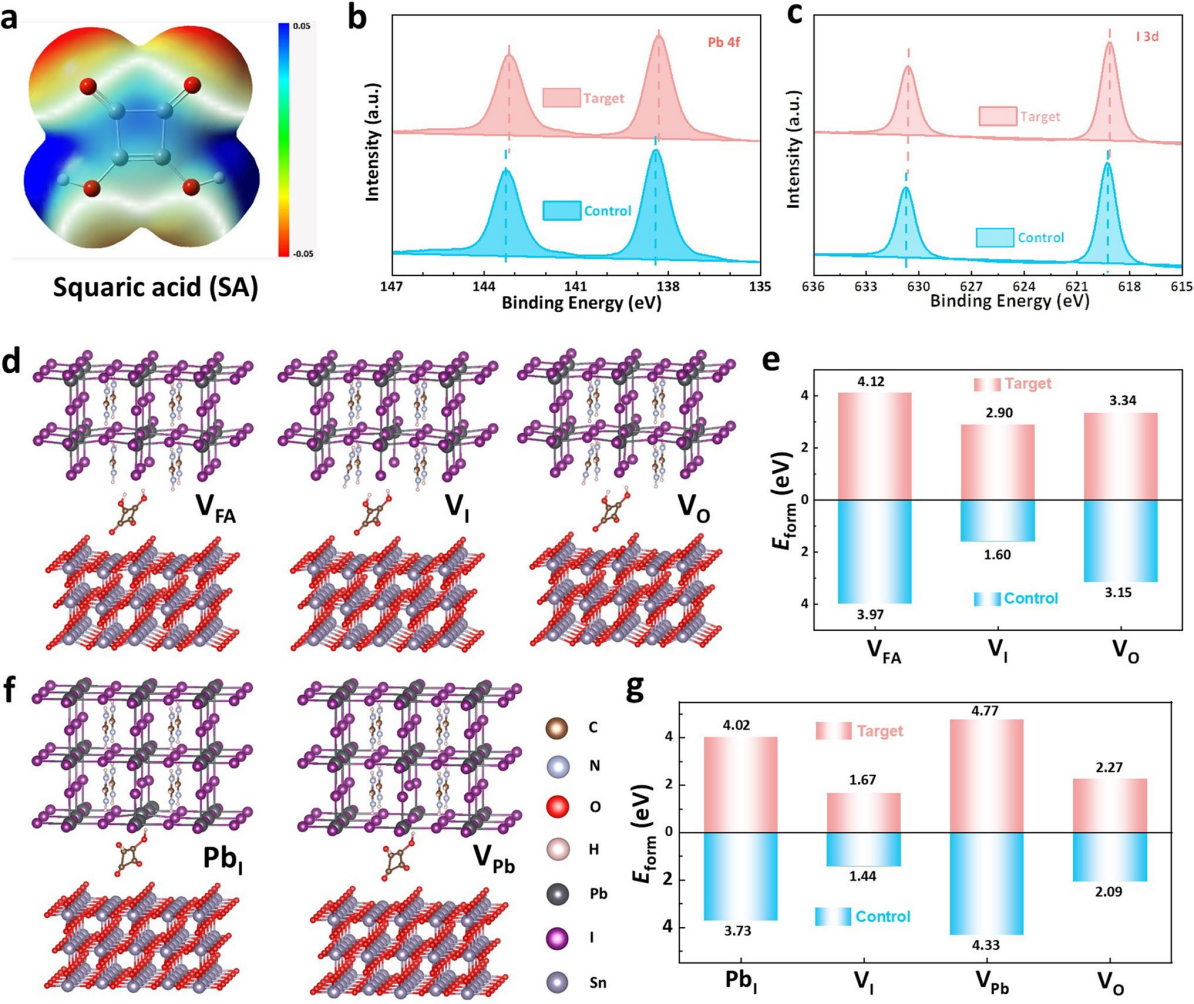
Chemical interaction and defect formation energy. **a** ESP profiles of SA. XPS spectra of **b** Pb 4f and **c** I 3*d* of the control and target perovskite films. **d** Theoretical models of V_FA_, V_I_ and V_O_ defect formation at the interface of SnO_2_/SA/ perovskite with the FAI-terminated surface. **e** *E*_form_ of V_FA_, V_I_ and V_O_ defect at the FAI-terminated surface for the control and target perovskite films. **f** Theoretical models of Pb_I_ and V_Pb_ defect formation at the interface of SnO_2_/SA/perovskite with the PbI_2_-terminated surface. **g** *E*_form_ of Pb_I_, V_I_, V_Pb_ and V_O_ defect at the PbI_2_-terminated surface for the control and target perovskite films

Herein, we introduced a new molecule, SA, to modify the buried interface. SA is a symmetrical planar biprotic quaternary carbon oxygen compound with unique 2π-quasi-aromatic properties and reversible self-transformation structures (Fig. S1), which can act as both a hydrogen bond donor and acceptor and an effective electron transport pathway. By DFT simulation, we obtained the electrostatic potential (ESP) of SA in Fig. 1a. There is a relatively positive electron cloud density at the H atom (blue region) and a relatively negative electron cloud density at the O atom (red and yellow region), indicating that the O atom is able to interact strongly with Pb^2+^ [33], passivating the defect while becoming the active site of perovskite crystallization. Furthermore, we studied the interaction of perovskite and SA by XPS. The characteristic signal of Pb 4*f* in the target perovskite film moves toward the low binding energy position (Fig. 1b), reflecting the Lewis acid base coordination interaction between the O atom in the SA and the undercoordinated Pb^2+^ in the perovskite film. In addition, the XPS peaks of I 3*d* (Fig. 1c) and N 1* s* (Fig. S2) show evident displacement, which are mainly caused by the strong hydrogen bond interaction between SA molecules and I^−^ and FA^+^/MA^+^ in perovskite.

Due to the special reversible self-transformation structures of SA, it can not only strongly interact with perovskite components to simultaneously passivate Pb-related defects, FA^+^/MA^+^ vacancy defects and I^−^/Br^−^ vacancy defects in perovskite, but also can effectively fill the V_O_ on the surface of SnO_2_ (Fig. S3). To verify the defect passivation effect of SA on SnO_2_ and perovskite, by DFT method (The detailed DFT calculation methods are provided in in Supporting Information), we calculated the formation energy (*E*_form_) of various defects at the interface between SnO_2_ and perovskite before and after the introduction of SA (Figs. 1d-g and S4-S6, Table S1). At the FAI-terminated surface, there are mainly FA vacancy (V_FA_), I vacancy (V_I_) and V_O_ defects. As shown in Figs. 1d, e and S4, the *E*_form_ of V_FA_, V_I_ and V_O_ in the reference system is calculated to be 3.97, 1.69, and 3.15 eV, respectively. In contrast, these values in target system increase to 4.12, 2.90, and 3.34 eV, respectively. At the PbI_2_-terminated surface, the *E*_form_ of the main defects for I-site substitution by Pb antisite defect (Pb_I_), Pb vacancy (V_Pb_), I vacancy (V_I_) and V_O_ are 4.02, 1.67, 4.77, and 2.27 eV (Figs. 1f, g and S5, S6), which is significantly higher than the defect *E*_form_ without SA [27, 34, 35]. The results show that SA has obvious passivation effect on various defects at the interface of SnO_2_ and perovskite.

### Interfacial Electron Transport and Photoelectric Performance of SnO_2_

**Fig. 2:**
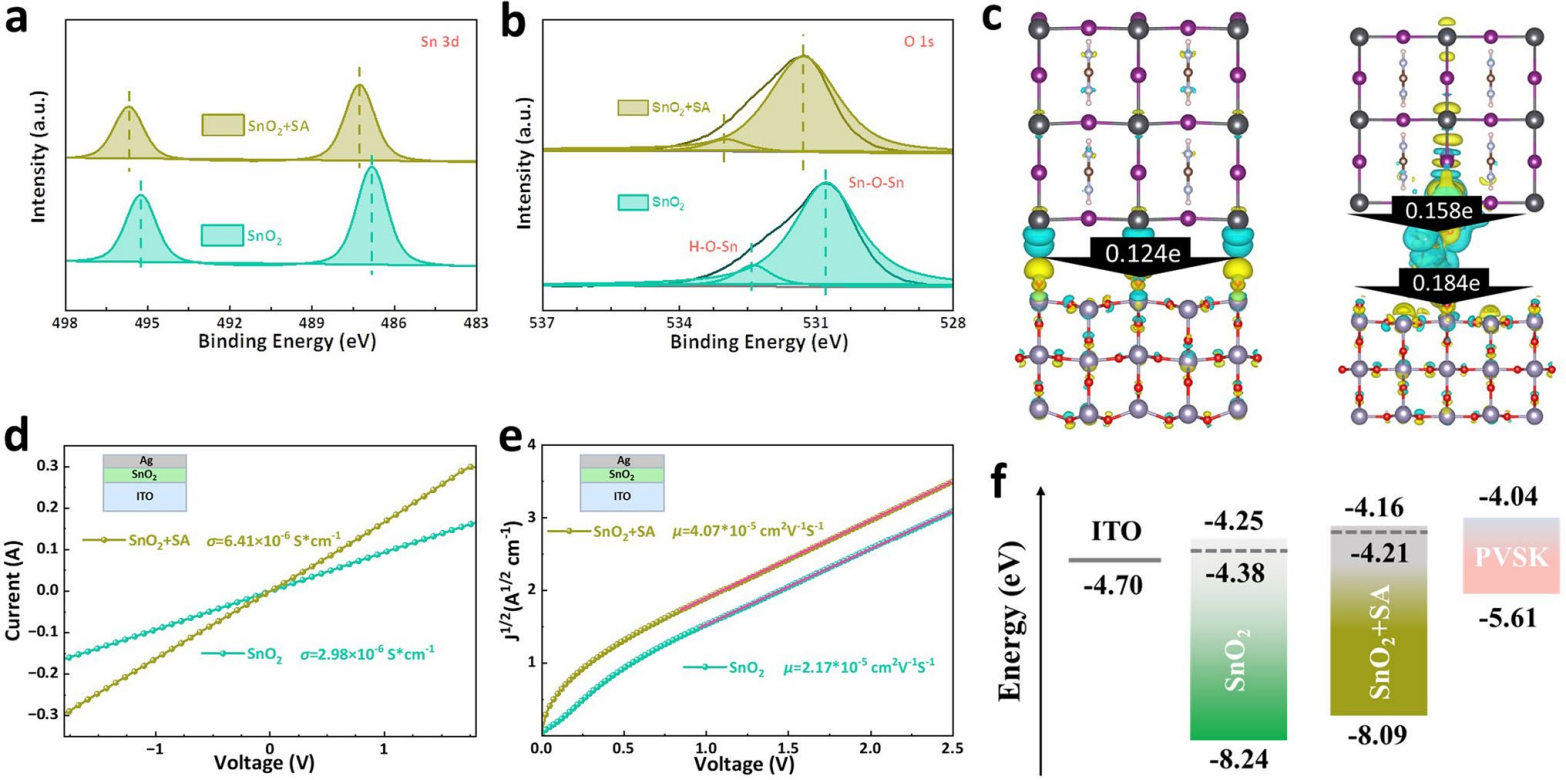
Influence of SA on charge transport and photoelectric properties of SnO_2_. XPS spectra of **a** Sn 3d and **b** O 1* s* for the SnO_2_ and SnO_2_ + SA films. **c** 3D charge density difference and Bader charge analysis at the buried SnO_2_/perovskite and SnO_2_/SA/perovskite interface. **d** Conductivity measurement of SnO_2_ and SnO_2_ + SA films. **e** Electron transport properties of the SnO_2_ and SnO_2_ + SA determined by the SCLC. **f** Schematic diagram of the energy level arrangement before and after SA modification

We compared XPS spectra to certify the effect of SA on the chemical environment of SnO_2_ layer. As can be seen from Fig. 2a, the Sn 3*d* peaks of the SnO_2_ film are, respectively, 495.28 and 486.78 eV, which shift to 495.68 and 487.28 eV for SA + SnO_2_, meaning that the decreased electron density after SA modification and the existence of SA on the surface of SnO_2_. In Fig. 2b, the O 1*s* signal of the control SnO_2_ film can be decoupled into two peaks of 530.78 and 532.38 eV, which is attributed to the lattice oxygen (Sn–O-Sn) and V_O_ on the surface of SnO_2_. In SA + SnO_2_ film, the two peaks shift to 531.28 and 532.98 eV and the peak intensity of V_O_ decreases, indicating that the V_O_ is occupied by O atom or OH of SA and the surface non-radiative recombination could be inhibited [21, 26]. The movement of Sn 3*d* and O 1*s* peaks reveals a strong chemical interaction between SA and SnO_2_ [26]. In addition, we also used FTIR spectroscopy to study the interaction between SA and SnO_2_. In the FTIR spectra (Fig. S7), after SA modification, the Sn–O-Sn bond displays evident blue shift from 558 (for SnO_2_) to 565 cm^−1^ (for SnO_2_ + SA). In SnO_2_ + SA sample, the stretching vibration peaks of = C–O–C, C-O, -C = O and out-of-plane bending vibration peak of -OH move from 632, 1049, 1166, and 1812 cm^−1^ to 640, 1057, 1172, and 1819 cm^−1^, respectively. These results all indicate the strong chemical interaction between SA and SnO_2_.

In addition, the reversible self-transforming structure of SA makes it become as an electron transport channel between perovskite and SnO_2_, allowing electrons to be transported rapidly through it (Fig. S1). To quantitatively assess charge transport at the SnO_2_/perovskite interface, the 3D charge density difference and average Bard charge were calculated using DFT, as shown in Fig. 2c. The 3D charge density difference visually illustrates the detailed nature of charge transfer of the SA at the buried SnO_2_/perovskite interface, where blue clouds represent electron losses relative to bonds or atoms after charge redistribution, and yellow clouds represent electrons captured relative to atoms [36–38]. It shows the prominent difference in charge density between the two interface structures, which is also reflected in the change in Bader charge. At the SnO_2_/perovskite interface, a charge of 0.124e is transferred from the perovskite to the SnO_2_ layer. In contrast, at the SnO_2_/SA/perovskite interface, the calculated charges of 0.158e and 0.184e are transferred from perovskite to SA and SA to SnO_2_, respectively [38, 39]. Both differential charge density and Bader charge analysis reveal that the introduction of SA leads to the remarkable difference in charge density at the SnO_2_/perovskite interface and SA as a charge transport channel can effectively promote carrier transfer.

Then, we studied the effect of SA on the photoelectric performance of SnO_2_. As can be seen from Fig. S8, the SA modification does not observably change the transmittance of SnO_2_ layer. Then, in Fig. 2d, the conductivity of SnO_2_ and SnO_2_ + SA ETLs was calculated using the formula: *σ* = *Id*/*VA*, where *A* is effective area, *d* is thickness of ETLs [40, 41]. The conductivity of SnO_2_ film with SA is slightly improved from 3.10 × 10^–6^ to 6.61 × 10^–6^ S cm^−1^, due to the presence of SA charge transfer molecular bridges and the strong chemical interaction between SA and SnO_2_. Next, in Fig. 2e, the electron mobility of SnO_2_ was measured using space charge-limited current (SCLC), and the curves are fitted according to the Mott-Gurney equation: *J* = 9*με*_0_*ε*_r_*V*^2^/(8*L*^3^) [41, 42]. The electron mobility of SnO_2_ + SA and SnO_2_ films are 5.88 × 10^–3^ and 3.22 × 10^–3^ cm^2^ V^−1^ s^−1^, respectively. The result further proves that SA modification enhances the electron transport of SnO_2_, which is in agreement with the theoretical calculation results in Fig. 2c. The band structure of SnO_2_ and SnO_2_ + SA was characterized by UPS, as shown in Figs. S9, S10, and Table S2. The band conduction minimum (CBM) of SnO_2_ and SnO_2_-SA ETL was derived from the valence band maximum (VBM), and the band gap (*E*_g_) values were determined by UV–vis absorption spectra and Tauc plots (Fig. S12). The CBM of SnO_2_ + SA is closer to the CBM of perovskite than that of the original SnO_2_ (Fig. 2f). A better alignment of energy levels between perovskite and SnO_2_ + SA helps avoid carrier build-up at the interface and increases *V*_oc_ [26, 43, 44].

### Crystallization and Morphology of Perovskite Films

**Fig. 3:**
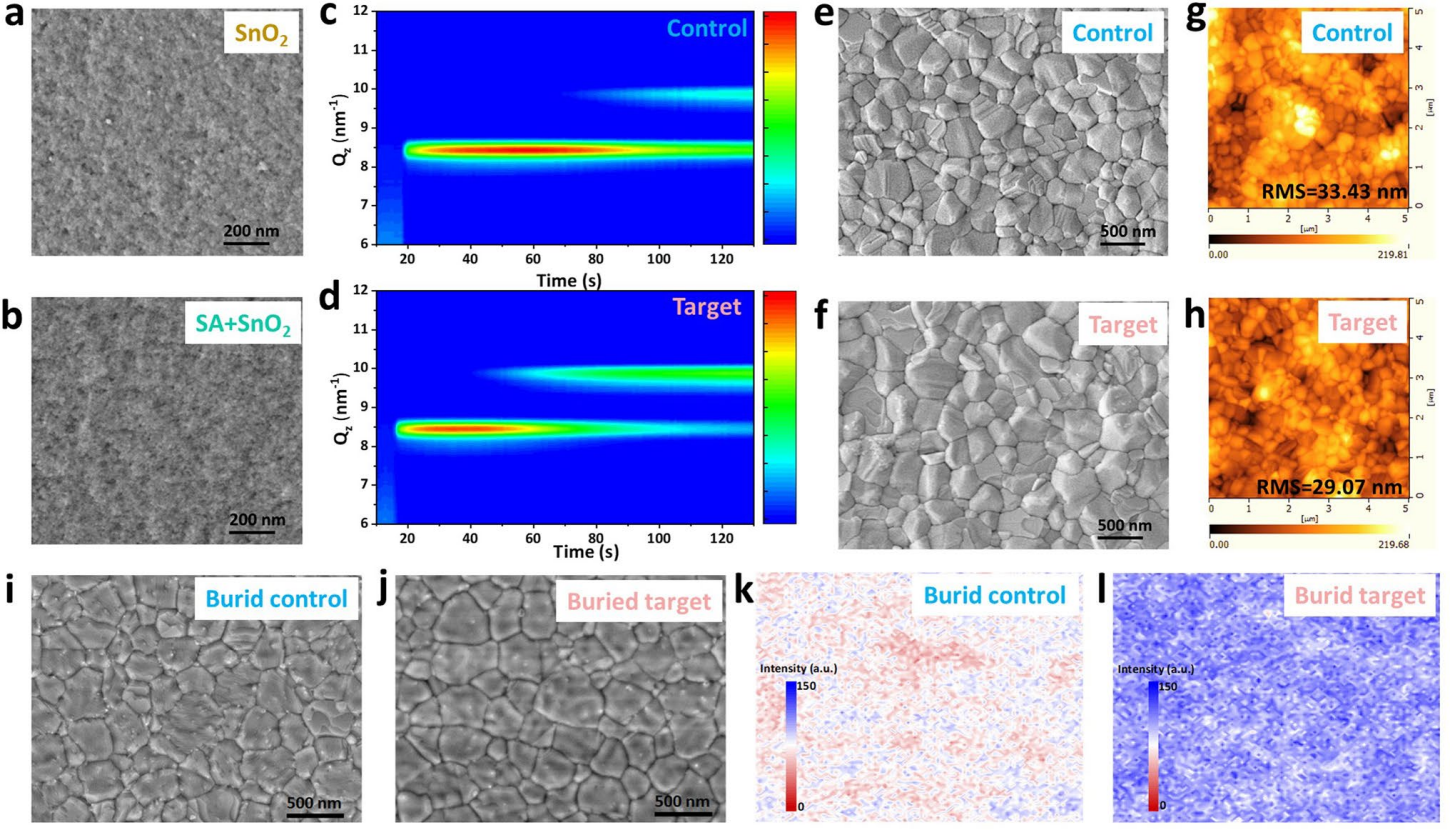
Influence of SA on crystallization and morphology of perovskite films. Top-view SEM images of **a** SnO_2_ and **b** SnO_2_ + SA films. 2D contour map obtained from the time-dependent in-situ GIWAXS patterns of the **c** control and **d** target perovskite films at the spin-coating stage. **e, f** Top-view SEM images and **g, h** AFM images of the control and target perovskite films. **i, j** Top-view SEM images and **k, l** PL mapping images of the buried interface from the bottom of the control and target perovskite films peeled off from SnO_2_ and SnO_2_ + SA

The surface morphologies of SnO_2_ before and after SA modification were firstly studied by top-view scanning electronic microscope (SEM) images. The original SnO_2_ nanoparticles are densely packed onto the ITO substrate (Fig. 3a). The deposition of SA obscures the GBs of these SnO_2_ nanoparticles, meaning that thin and continuous SA layer is successfully deposited on SnO_2_ layer (Fig. 3b). Furthermore, to investigate the role of SA in the crystallization of perovskite films, the time-dependent in-situ GIWAXS patterns was performed at the spin-coating stage. As shown in Fig. 3c, d, at the instant of antisolvent drops (approximately 18 s), the perovskite film on the SA-modified SnO_2_ layer exhibits a stronger δ-phase FA perovskite signal (Qz ≈8.34 nm^−1^) than the control film. In the subsequent process, compared with the control sample, the target perovskite film dispalys quickly appeared α phase FA perovskite signal (Qz ≈9.91 nm^−1^) at about 45 s along with the continuedly increased intensity, meanwhile, the δ phase FA peak gradually decreases [45, 46]. The results indicatie that δ phase FA perovskite is transformed into α phase FA perovskite more quickly, which means that SA is beneficial to lead a more effective and faster phase transition process to reduce the hidden danger caused by the difficulty of converting mesophase crystals into photoactive phases [47, 48].

From Figs. 3e, f and S13, it can be clearly seen that the perovskite grown on the SA-modified SnO_2_ layer has a better morphology with more uniform, reduced GBs and larger grain sizes (Fig. S14). The improved crystallinity of the target perovskite film induced by SA stems from strong interactions between SA and perovskite, which can reduce the number of nucleation centers and lead to the formation of a uniform crystalline structure with larger grain sizes. In addition, the root mean square (RMS) roughness of the perovskite films before and after modification was investigated by atomic force microscopy (AFM) measurements (Fig. 3g, h). The RMS of the control and target films is 33.43 and 29.07 nm, respectively. The reduced roughness also indicates that SA is conducive to uniform nucleation and crystallization of perovskite film.

Subsequently, to further evaluate the influence of the buried interface with and without SA on the growth of perovskite, the morphology and PL properties of the buried interface were studied by SEM images and PL mapping tests. The results (Fig. 3i, j) are consistent with those measured by top-view SEM images (Fig. 3e, f). The perovskite film grown on SnO_2_ + SA displays denser structure with larger grain size and smaller grain boundaries. In contrast, the SnO_2_-based perovskite film has more irregular crystals. As shown in Fig. 3k, l, in comparison with the buried control film, the buried target perovskite film displays observably enhanced overall fluorescence localization intensity with more uniform distribution, indicating that the significantly suppressed non-radiative recombination caused by surface defects. These results indicate that the SA-treated SnO_2_ layer can promote the growth of perovskite crystals, finally to obtain high quality and low defect target film.

### Carrier Dynamics and Residual Stress Investigation

**Fig. 4:**
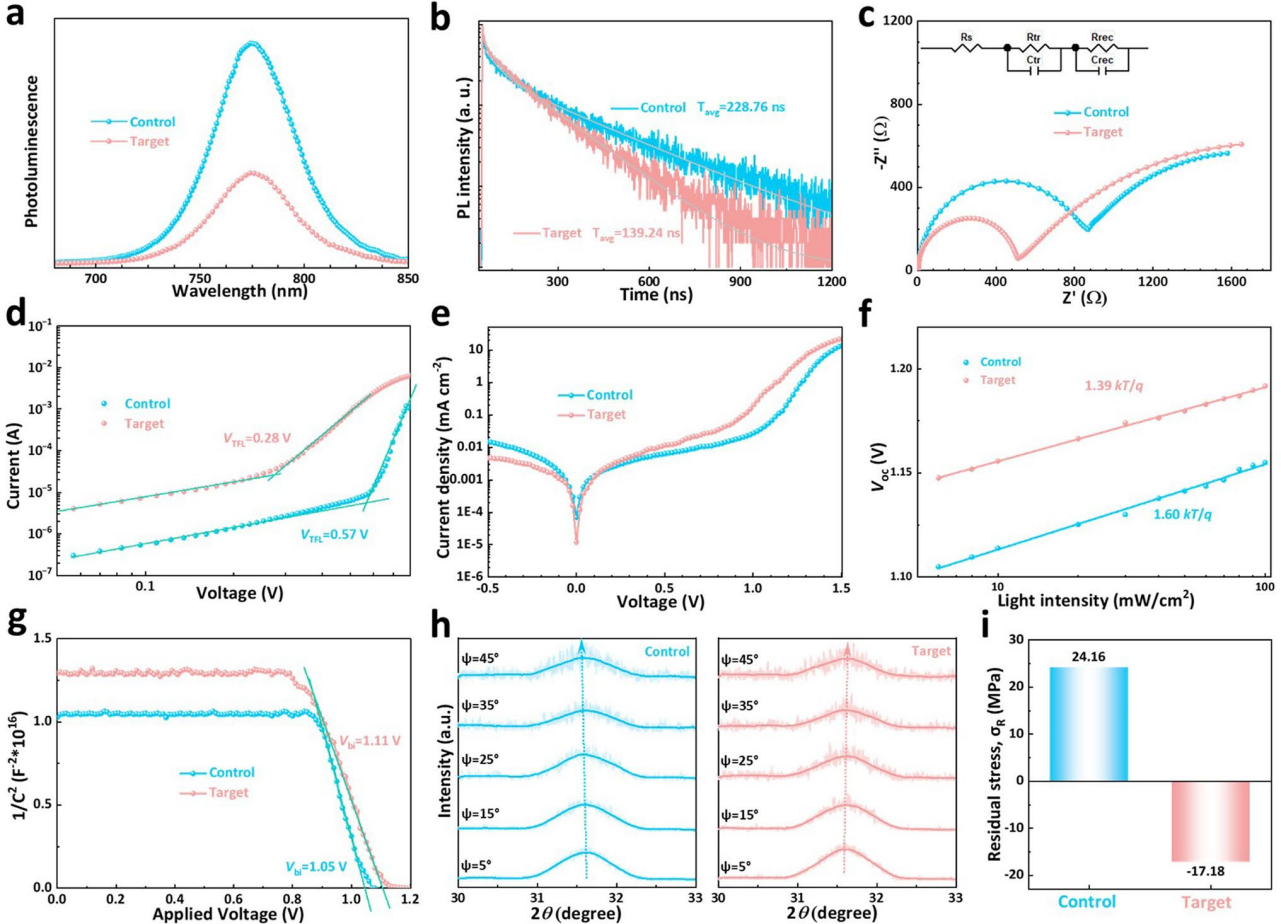
Carrier Dynamics and Residual Stress of perovskite films and devices. **a** Steady-state PL spectra and **b** TRPL spectra of the control and target perovskite/SnO_2_/ITO without and with SA. **c** Nyquist plots at *V* = -1.0 V, **d** Dark current–voltage (*I-V*) curves with the configurations of electron-only (glass/ITO/SnO_2_/perovskite/passivation layer/PCBM/Au), **e** Dark *J-V* curves, **f** *V*_oc_ values of the corresponding devices versus light intensity on a seminatural logarithmic scale and **g** Mott-Schottky plots of the control and target perovskite devices. **h** GIXRD patterns measured at different *ψ* angles from 5° to 45° and **i** comparison of residual stress for the control and target perovskite films

The quality of perovskite layer with perovskite/SnO_2_/glass structure was further studied by using XRD patterns and UV–vis absorption spectra. As can be seen in Figs. S16 and S17, and Table S3, the SA-modified perovskite film displays higher diffraction peak intensities and smaller full width at half maximum (FWHM) values for the (110) lattice planes, meaning that the SA-modified SnO_2_ is conducive to the growth of perovskite crystals, resulting in increased crystallinity and improved film quality. In Fig. S18, the slightly increased absorption intensity in the UV–vis absorption spectra predicts that the quality of the target perovskite film is higher. Furthermore, the charge transport behavior of the perovskite films on the SnO_2_ without and with SA studied by using steady-state PL spectra and time-resolved TRPL spectra. According to PL spectra in Fig. 4a, the PL intensity of the perovskite layer after SA modification is significantly reduced, indicating that SA not only passivates the interface to promote perovskite crystallization but also facilitates the electron transport between SnO_2_ and perovskite, endowing it with higher film quality, lower defect density and reduced non-radiation recombination. TRPL spectra in Fig. 4b were recorded to quantitatively compare the charge transfer of the control and target perovskite films. The average PL decay lifetime of the target perovskite film reduces from 228.76 ns for control film to 139.24 ns. The shorter average PL decay lifetime indicates a much faster charge transfer process from perovskite to SnO_2_, which is mainly caused by the greatly reduced defect density in target perovskite film and is likely to contribute to better photovoltaic performance.

We collected the charge transfer resistance (*R*_ct_) and carrier recombination resistance (*R*_rec_) in the device by EIS under dark conditions with an applied voltage of −1.0 V in Fig. 4c. Compared with the control device, the *R*_ct_ of the target device is significantly reduced from 892.46 to 516.01 Ω and the *R*_rec_ is significantly increased from 1511.54 to 2323.42 Ω, indicating that the target device has a faster charge transfer rate and a slower carrier recombination rate. The trap density of perovskite device is calculated by space charge current limiting (SCLC) technique. The electron-only devices with the structure of ITO/SnO_2_/perovskite/PCBM/Au were prepared. The corresponding dark *J-V* curves are exhibited in Fig. 4d, and the trap-filling voltage (*V*_TFL_) can be obtained. *V*_TFL_ of the devices before and after treatment are 0.57 and 0.28 V, respectively. The trap density (*N*_t_) was calculated from *N*_t_ = 2*V*_TFL_*ε*_0_*ε*_r_/(*qL*^2^). The *N*_t_ of the control and target perovskite devices were 4.75 × 10^15^ and 1.97 × 10^15^ cm^−3^, respectively. The results show that the defects of target perovskite are effectively suppressed owing to its higher film quality.

Dark *J-V* curves (Fig. 4e) were measured to further investigate the charge transfer and recombination behavior of PSCs. The reverse leakage current density and dark saturation current density (*J*_0_) of the target perovskite device are smaller, indicating that the reduced bulk defect, improved charge extraction, effectively inhibited charge recombination and increased *V*_oc_ [49]. The result is consistent with the EIS results of a decrease in *R*_tr_ and an increase in *R*_rec_. The dependence of light intensity on *J*_sc_ and *V*_oc_ has also been used to investigate the effect of SA on charge extraction and defect passivation of target perovskites. As shown in Fig. S19, the α value of the target perovskite device (0.997) is closer to 1 than that of the control device (0.918), implying the fast separation of the photogenerated electron–hole pairs and inhibited trap-assisted carrier recombination in the target perovskite device, due to the better energy level matching between SA + SnO_2_ and perovskite. Figure 4f shows the logarithmic relationship of *V*_oc_ with light intensity, where the slope of the line is n *kB*/*T*. When n value is greater than and close to 1, which means the appearance of defect assisted recombination and reduction of defect assisted recombination in the PSCs, respectively. It can be seen from Fig. 4f that the n value of the target perovskite device is significantly reduced, which proves that the target perovskite device displays fewer defects, thus reducing the non-radiative recombination and increasing the *V*_oc_. Mott-Schottky plots were used to evaluate the improvement of *V*_oc_ and built-in potential (*V*_bi_) in perovskite devices. In Fig. 4g, the *V*_bi_ values of the control and target perovskite devices are 1.05 and 1.11 V, respectively. The target device exhibits higher *V*_bi_, indicating that the built-in band alignment on heterogeneous structures, which makes the more efficient carrier collection at the perovskite/ETL interface [50].

The SA has beneficial impacts on the morphology and crystallinity of perovskite, which also influence the release of residual stress. Therefore, we investigated the residual stress of the perovskite films using GIXRD (Fig. 4h). The results show that the diffraction peaks of the control perovskite gradually shift to lower 2*θ* positions as *ψ* changes from 5 to 45°, whereas the diffraction peaks of the target perovskite slightly move to higher 2*θ* positions. According to Bragg’s Law and generalized Hooke’s Law, the relationship between 2*θ* and sin^2^*ψ* can be expressed by the following equation: σ =  − *E*_*p*_πcot*θ*_0_/[2(1 + *ν*_*p*_)180°]*∂(2*θ*)/∂[(sin^2^*ψ*)] (*E*_p_ is the perovskite modulus (10 GPa), *ν*_p_ is Poisson’s ratio of the perovskite (0.3) and *θ*_0_ is half of the scattering angle 2*θ*_0_ for stress-free perovskite (2*θ*_0_ = 31.6°)) [51]. By fitting 2*θ* as a function of sin^2^*ψ* (Fig. S20), the residual stress in the perovskite films can be calculated using the above equation. The slope of the fitted line indicates the magnitude of residual strain. Negative slope means that the film endures tensile stress, whereas positive slope represents the film undergoes compressive stress [51–53]. The calculated results are shown in Fig. 4i, revealing that the control perovskite subjects a tensile stress of 24.6 MPa. In contrast, the target perovskite film exhibits a slight compressive stress (−17.18 MPa). It indicates that the introduction of SA can release the residual tensile stress in the perovskite film, which is beneficial for improving the PCE and stability of PSCs [53].

### Photovoltaic Performance and Stability

**Fig. 5:**
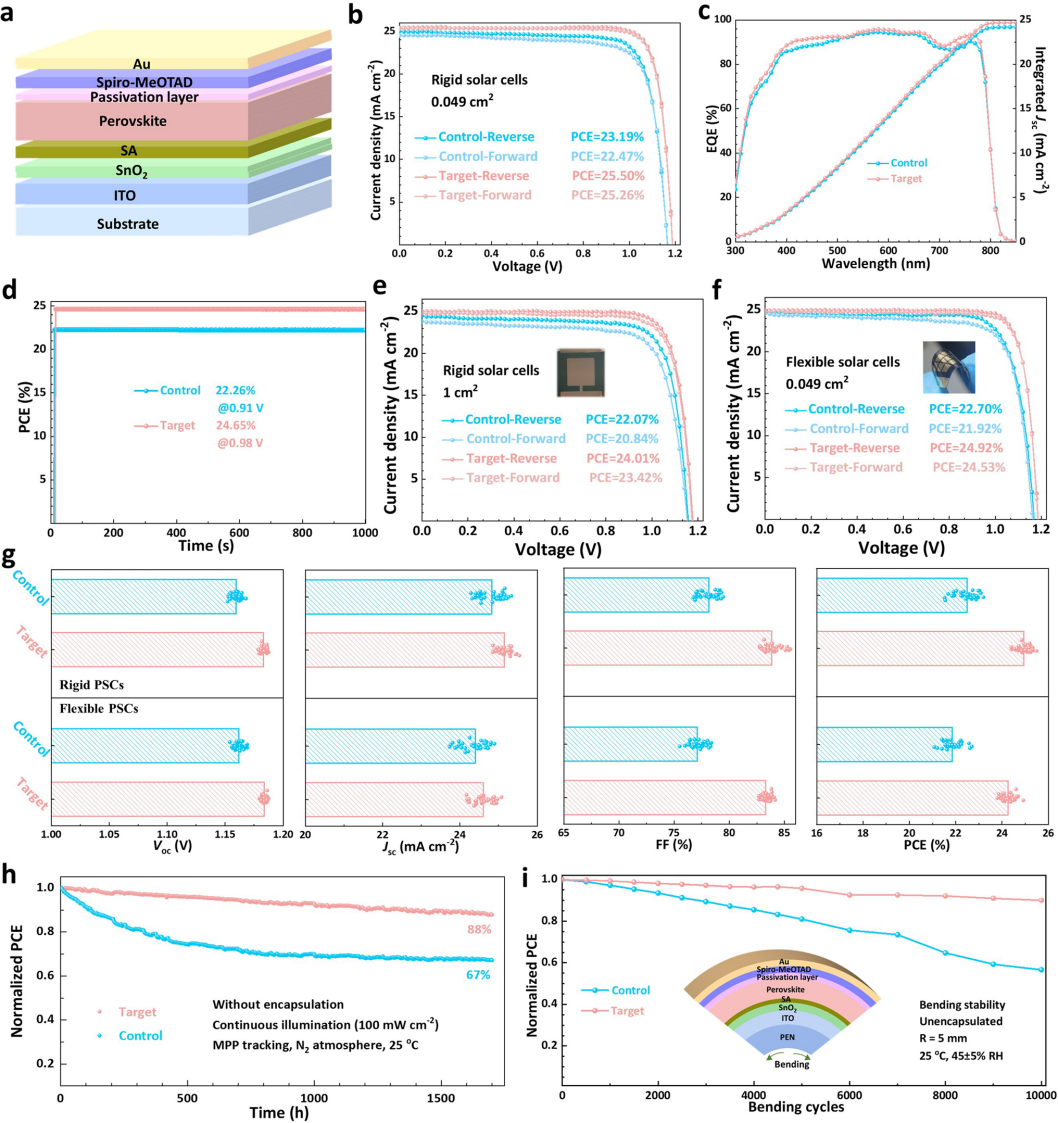
Photovoltaic performance and stability of PSCs. **a** Schematic architecture of the fabricated PSCs. **b** *J-V* curves under reverse and forward scan directions, **c** EQE spectra and **d** stabilized PCE at maximum power point as a function of time of the control and target perovskite devices. **e** *J-V* curves of the large-area rigid PSCs (1 cm^2^) and **f** *J-V* curves of flexible PSCs under reverse and forward scan directions. **g** Statistics of the photovoltaic parameters for the control and target rigid and flexible PSCs. **h** Normalized PCE of the unencapsulated control and target PSCs measured at MPP under continuous 1-sun illumination in ambient atmosphere and at room temperature. **i** Normalized PCE variation curves of the unencapsulated control and target flexible PSCs over the bending cycle at R = 5 mm

Based on the structure of ITO/SnO_2_/perovskite/passivation layer/spiro-OMeTAD/Au (Fig. 5a), we investigated the effect of the introduction of SA on photovoltaic performance of PSCs. The optimized *J-V* curve under reverse and forward scan directions with a scan speed of 0.05 V s^−1^ of the champion devices (When the concentration of SA is 5 mg mL^−1^) and the corresponding photovoltaic parameters are shown in Fig. 5b and Table S6. The champion PCE of target devices improves significantly from 23.19% of control devices to 25.50%, mainly due to the increase in *V*_oc_ (from 1.17 to 1.19 V), *J*_sc_ improvement (from 25.04 to 25.47 mA cm^−2^) and FF improvement (from 79.46 to 84.30%). After calculation, the target device also exhibits a lower hysteretic index than the control device, declining from 3.10% (for control) to 0.94%, which may be caused by the simultaneous reduced the bulk and interface to effectively prevent ion diffusion [54].

Figure 5c displays the EQE spectra of the corresponding PSCs, demonstrating the superior performance of the target PSC in enhancing *J*_sc_. Furthermore, to investigate the delay and reproducibility of the PSCs, we monitored both the stabilized power output (SPO) at maximum *V*_oc_ (*V*_m_) over 1000 s under continuous one-sun illumination (Fig. 5d). To further verify the effective role of SA, we also fabricated large-area perovskite devices and flexible PSCs and the corresponding *J-V* curves are shown in Fig. 5e, f. The large-area rigid PSC (1 cm^2^) based on SA yields a PCE of 24.01% and exhibits significantly reduced hysteresis (Fig. 5e). The target flexible PSC (0.049 cm^2^) delivers a considerable PCE of 24.92%, which is much higher than that of control device (22.70%). Moreover, the hysteresis index analysis reveals a decrease from 3.44% (control) to 1.57% for the target flexible device. Figure 5g shows the statistical photovoltaic parameters for the control and target rigid and flexible PSCs and the target rigid and flexible PSCs demonstrate better reproducibility and higher average photovoltaic parameters. The results show that SA contributes to an overall enhancement in *V*_oc_, *J*_sc_ and FF of both rigid and flexible devices, and thus improving PCE. These outstanding photovoltaic properties highlight the potential of SA as a multifunctional interlayer that can be widely used to manufacture highly efficient PSCs.

Subsequently, we verified the long-term stability of perovskite devices at 45 ± 5% RH and 85 °C, respectively (Figs. S23-S29). Firstly, we observed the actual morphological changes of target perovskite film, all perovskite films were placed in a moisture-aged environment (Fig. S23). The control film gradually changes from black to brown to pale yellow, showing serious degradation. In contrast, there is few noticeable differences in the target film, indicating its excellent moisture resistance due to reduced bulk and surface defect. Then, we performed water contact angle measurements to verify the high hydrophobic property of target perovskite film (Fig. S24). It can be seen that the contact angle of the target perovskite film (90.6°) is much larger than that of the control film (75.1°), indicating its better hydrophobicity. Figure S25 also shows the humidity stability of unsealed PSCs under 45 ± 5% RH at room temperature. After 3840 h of aging, the PCE of the control PSC decreases to 64% of the original value, while the target PSC still retains more than 93% of the initial PCE. Figure S27 examines the thermal stability of unencapsulated PSCs under 20% RH at 85 °C. The target device maintains about 88% of their initial PCE after 528 h, in contrast, the PCE of control device drops to 57% of the original value. We also compared the light absorption intensity of the perovskite films during thermal aging. As shown in Fig. S29, with the progress of temperature aging, the absorption intensity of the control film decreases rapidly, while the decline rate of the target film is significantly lower, which can maintain about 93% of its initial absorption intensity after 500 h of aging at 85 °C, suggesting that it has better thermal resistance.

In addition, more rigorous stability measurements were conducted on both control and target perovskite devices. The operational stability of unencapsulated devices was evaluated under continuous 1-sun illumination at maximum power point tracking (MPPT) in a nitrogen (N_2_) atmosphere at room temperature. The corresponding changes in PCE are shown in Fig. 5h. It can be observed that the control device exhibits significant PCE degradation, retaining only 67% of its initial PCE after 1700 h. In contrast, the target device demonstrates markedly enhanced operational stability, and maintains 88% of its original PCE after the same period. Figure 5i presents the results of bending stability tests for the control and target PSCs under ambient conditions. With a bending radius (R) of 5 mm, the PCE of the control device rapidly decreases as the number of bending cycles increases, dropping to 56% of its initial PCE after 10,000 cycles. Whereas, the target perovskite device presents enhanced mechanical stability, maintaining over 90% of its original PCE after the same number of bending cycles. The overall stability enhancement of the target perovskite device primarily stems from the role of SA in improving perovskite film quality, reducing defects, suppressing ion migration, strengthening interfacial connections, and dynamically releasing residual stress under external environmental stress. This further validates the advantages of the self-management bilateral bonding effect of SA.

## Conclusions

In summary, SA, due to its unique structure, exhibits self-managed bilateral bonding characteristics, enabling comprehensive modulation of the buried interface between SnO_2_ and the perovskite layer, which significantly enhances the PCE and stability of PSCs. Firstly, SA interacts with SnO_2_, Pb^2+^, FA^+^/MA^+^, and I^−^/Br^−^, effectively filling surface oxygen vacancies and passivating defects in the perovskite layer, thereby improving the interface quality. Secondly, while adjusting the energy levels of SnO_2_, SA serves as a charge transport channel through structural self-transformation, which facilitat the transfer of electrons from the perovskite to the SnO_2_ ETL, thereby improving interfacial charge transfer and reducing carrier recombination. Finally, SA acts as a nucleation center to regulate the crystal growth of the perovskite, resulting in high-quality perovskite films with increased grain size and preferential orientation. Consequently, the SA-modified PSCs demonstrate significant improvements in *J*_sc_, *V*_oc_, and FF, achieving a high PCE of 25.50%. Notably, large-area rigid (1 cm^2^) and flexible devices yield excellent PCEs of 24.01% and 24.92%, respectively. Additionally, the unencapsulated SA-modified devices display excellent stability under environmental stress, including humidity, thermal aging, light irradiation, and bending conditions. This study provides valuable insights into the rational design of multifunctional molecules with self-transformation properties for optimizing the buried interface of perovskite materials, paving the way for their practical application and commercialization in highly efficient and stable PSCs.

## Supplementary Information

Below is the link to the electronic supplementary material.

Supplementary file1 (DOCX 5663 KB)
