# Supplementary material for: Self-Regulated Bilateral Anchoring Enables Efficient Charge Transport Pathways for High-Performance Rigid and Flexible Perovskite Solar Cells
Source: Nanomicro Lett. 2025 Jul 14;17:328. doi: 10.1007/s40820-025-01846-6 (PMC12259523; doi:10.1007/s40820-025-01846-6)
Supplement: Supplementary file 1 — Supplementary file1 (DOCX 5663 KB) [file 40820_2025_1846_MOESM1_ESM.docx]

Supporting Information for

Self-Regulated Bilateral Anchoring Enables Efficient Charge Transport Pathways for High-Performance Rigid and Flexible Perovskite Solar Cells

Haiying Zheng^1, 4^, Guozhen Liu^2,^ *, Xinhe Dong^4^, Feifan Chen^4^, Chao Wang^4^, Hongbo Yu^1^, Zhihua

Zhang^1,^ * and Xu Pan^3,^ *

^1^ School of Materials Science and Engineering, Dalian Jiaotong University, Dalian 116028, P. R. China

^2^ State Key Laboratory of Fine Chemicals, School of Chemistry, Dalian University of Technology, Dalian 116024, P. R. China

^3^ Key Laboratory of Photovoltaic and Energy Conservation Materials, Institute of Solid State Physics, Hefei Institutes of Physical Science, Chinese Academy of Sciences, Hefei 230031, P. R. China

^4^ Institutes of Physical Science and Information Technology, Anhui University, Hefei 230601, P. R. China

*Corresponding authors. E-mail: [gzliu@dlut.edu.cn](mailto:gzliu@dlut.edu.cn) (Guozhen Liu); [zhzhang@djtu.edu.cn](mailto:zhzhang@djtu.edu.cn) (Zhihua Zhang); [xpan@rntek.cas.cn](mailto:xpan@rntek.cas.cn) (Xu Pan)

**Supplementary Figures**


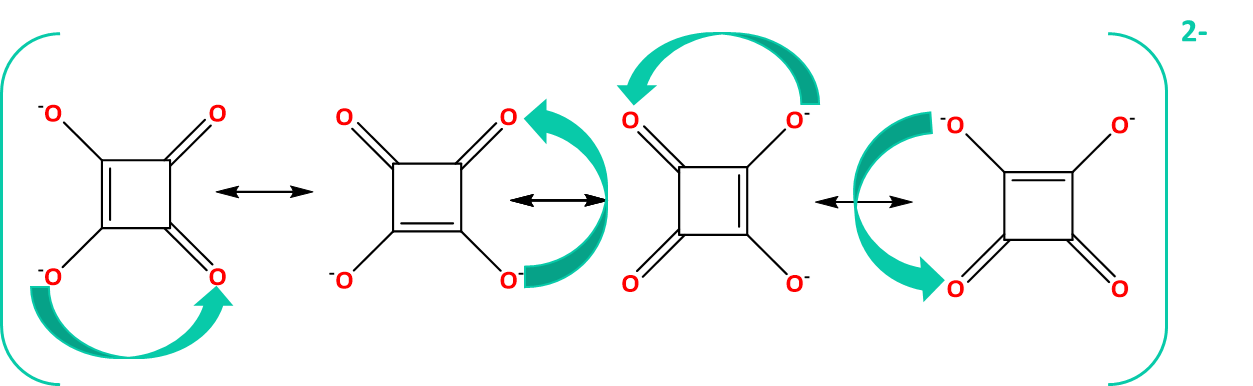


**Fig. S1** Reversible self-transformation structures and electron transfer process of SA

**Fig. S2** XPS spectra of N 1s for the control and target perovskite films


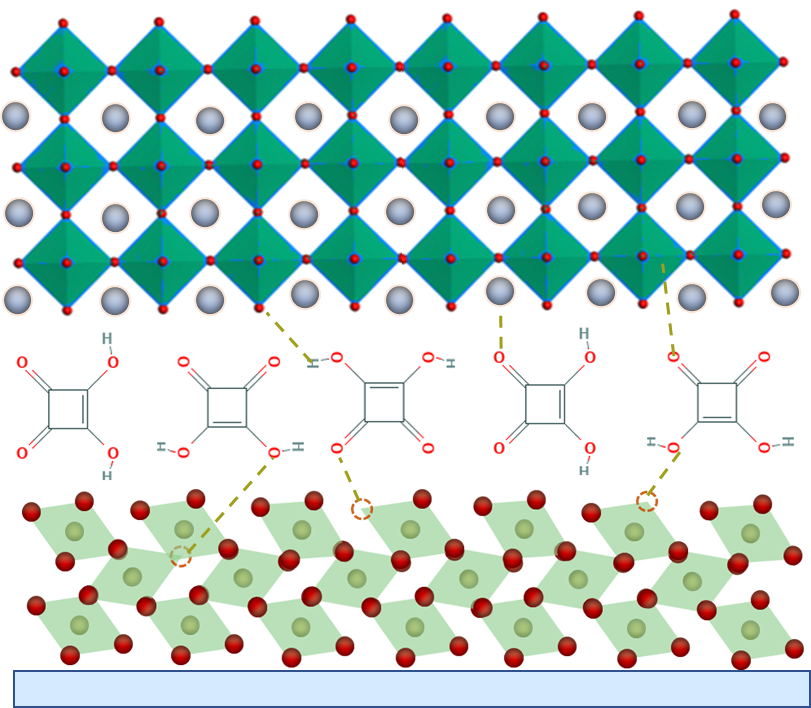


**Fig. S3** Schematic diagram for the passivation function of SA at the buried SnO_2_/perovskite interface


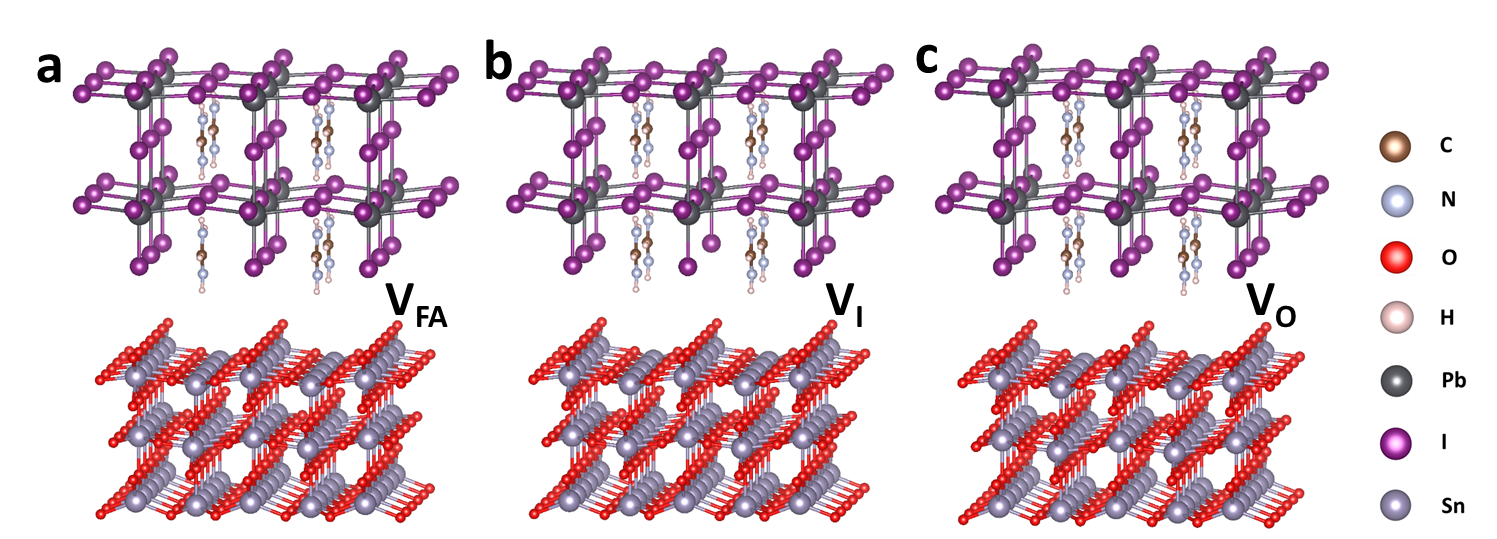


**Fig. S4** Theoretical models of **a** V_FA_, **b** V_I_ and **c** V_O_ defect formation at the interface of SnO_2_/perovskite with the FAI-terminated surface


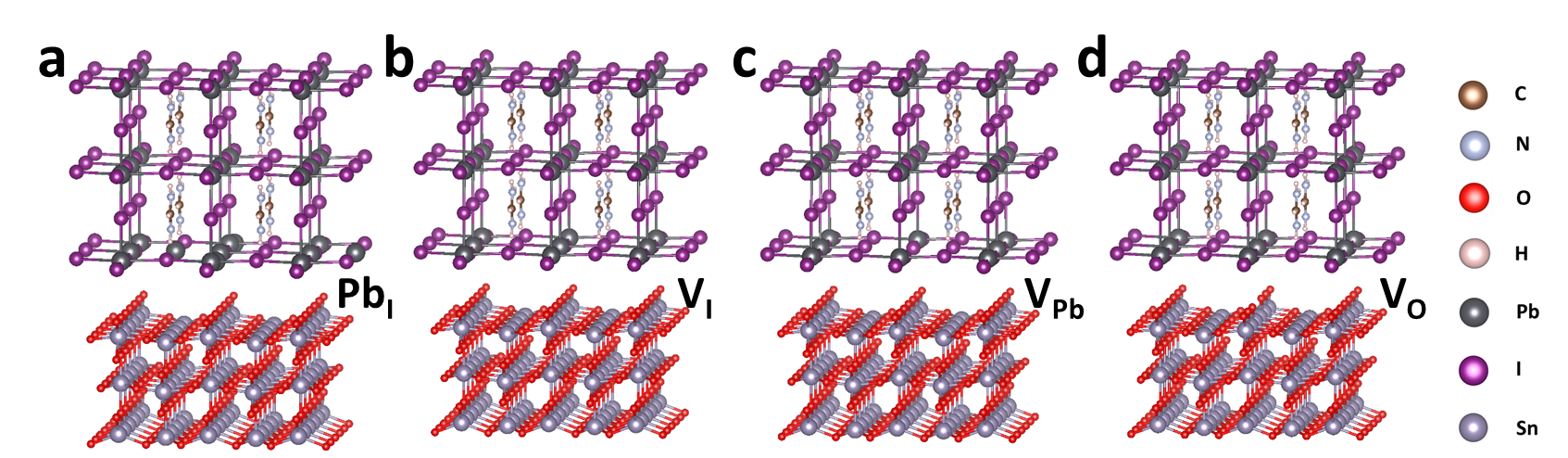


**Fig. S5** Theoretical models of **a** Pb_I_, **b** V_I_, **c** V_Pb_ and **d** V_O_ defect formation at the interface of SnO_2_/perovskite with the PbI_2_-terminated surface


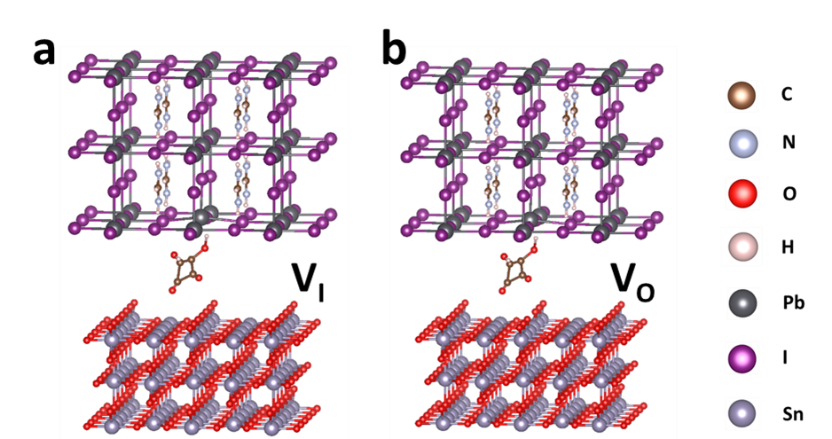


**Fig. S6** Theoretical models of **a** V_I_ and **b** V_O_ defect formation at the interface of SnO_2_/SA/ perovskite with the PbI_2_-terminated surface

**Fig. S7** FTIR spectra of SA, SnO_2_ and SnO_2_+SA

**Fig. S8** The transmittance spectra of the SnO_2_ and SnO_2_+SA films

**Fig. S9** UPS spectra in the region of *E*_cutoff_ binding energy of SnO_2_ and SnO_2_+SA


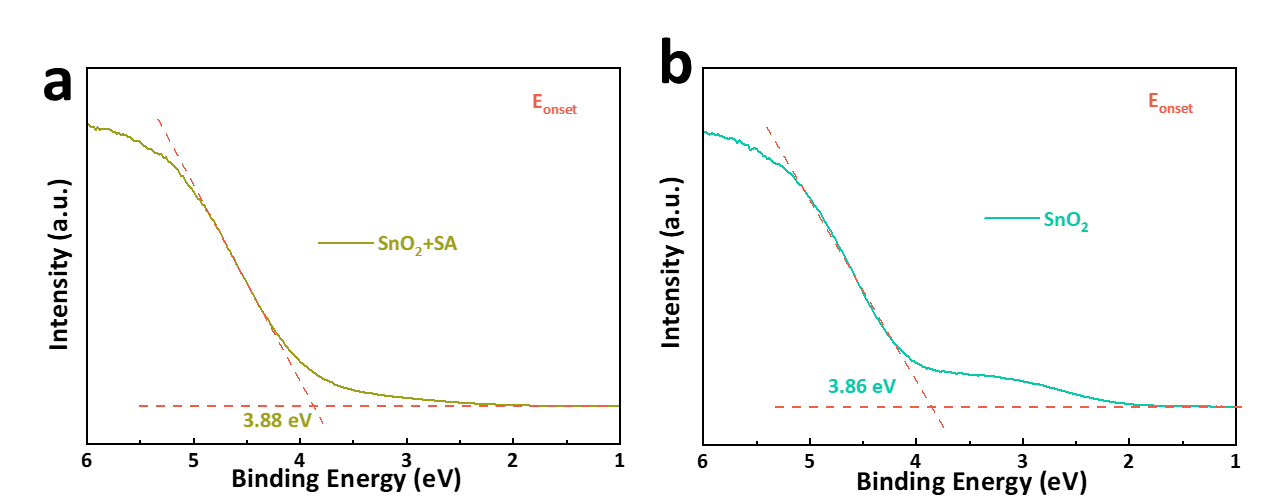


**Fig. S10** UPS spectra of *E*_onset_ binding energy of **a** SnO_2_ and **b** SnO_2_+SA


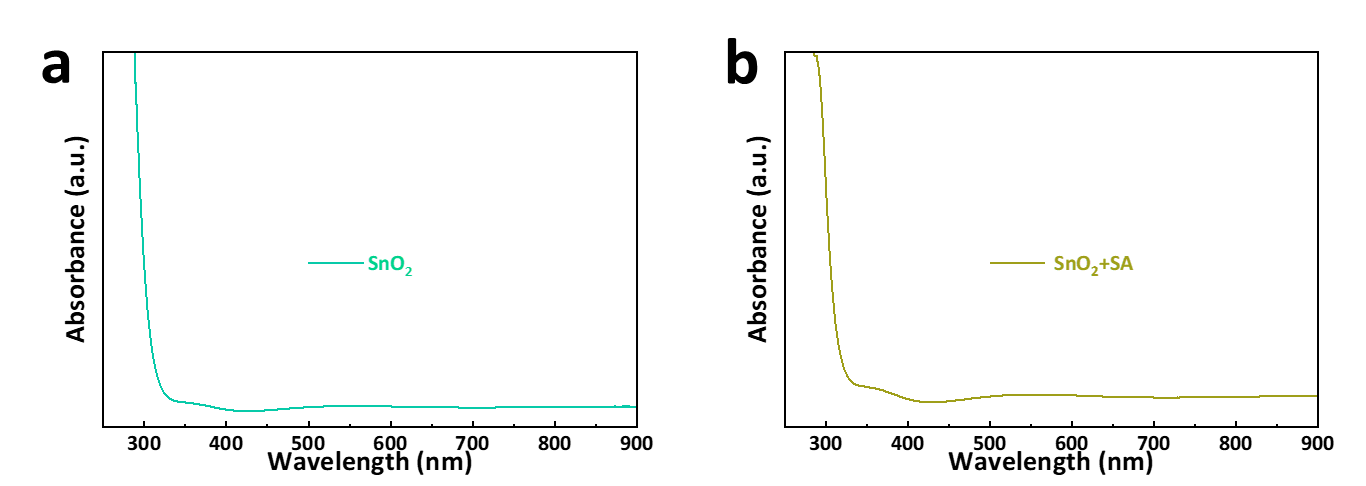


**Fig. S11** UV-vis absorption spectra of **a** SnO_2_ and **b** SnO_2_+SA films


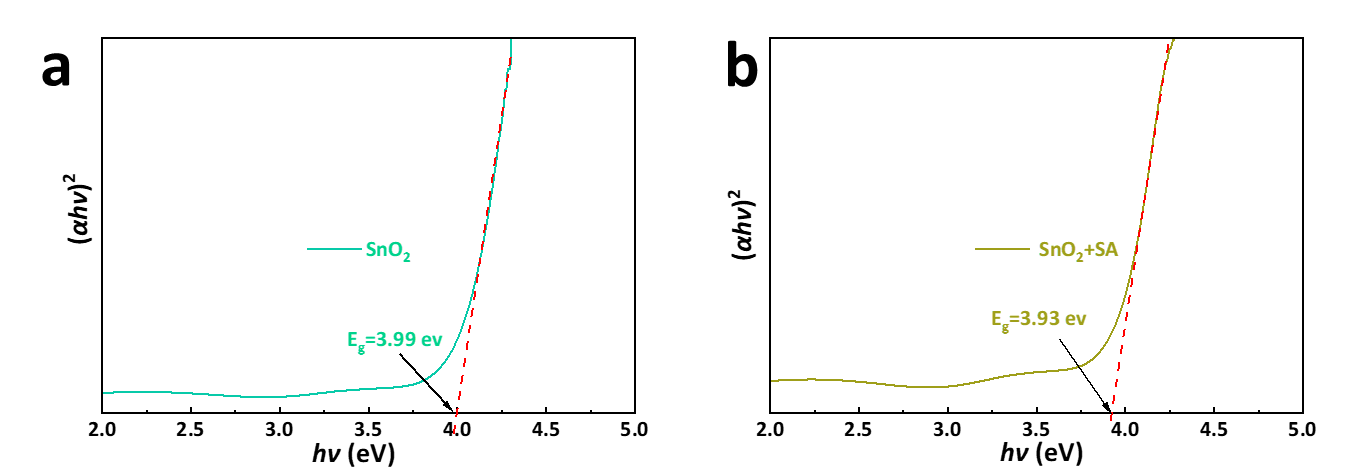


**Fig. S12** Tauc plots of **a** SnO_2_ and **b** SnO_2_+SA films


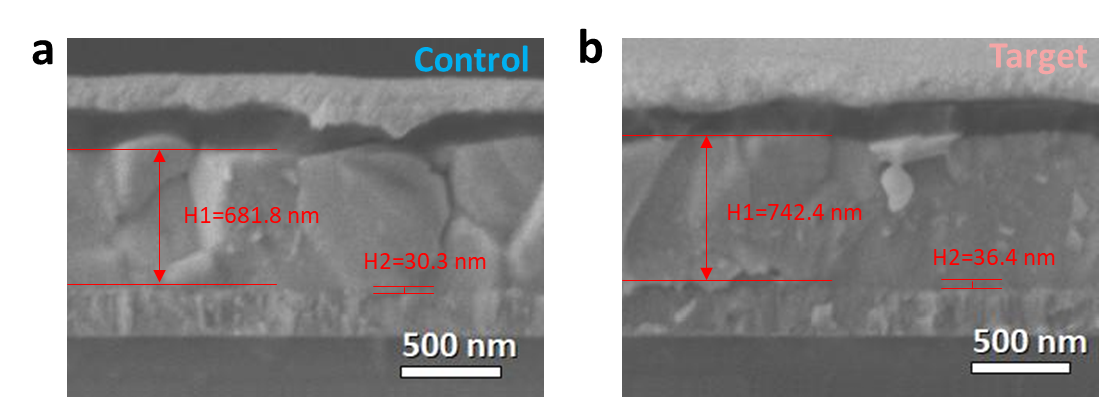


**Fig. S13** Cross-sectional SEM images of **a** control and **b** target perovskite devices


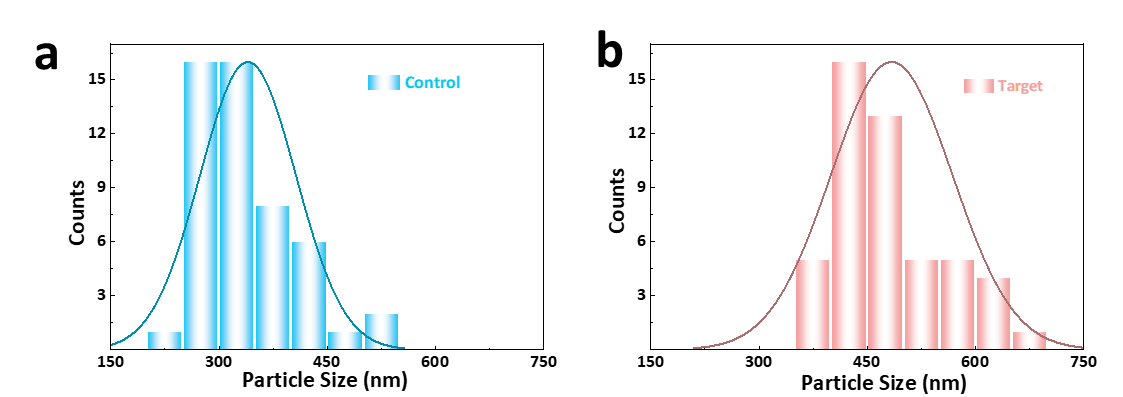


**Fig. S14** Grain size statistics of **a** control and **b** target perovskite films from top-view SEM images


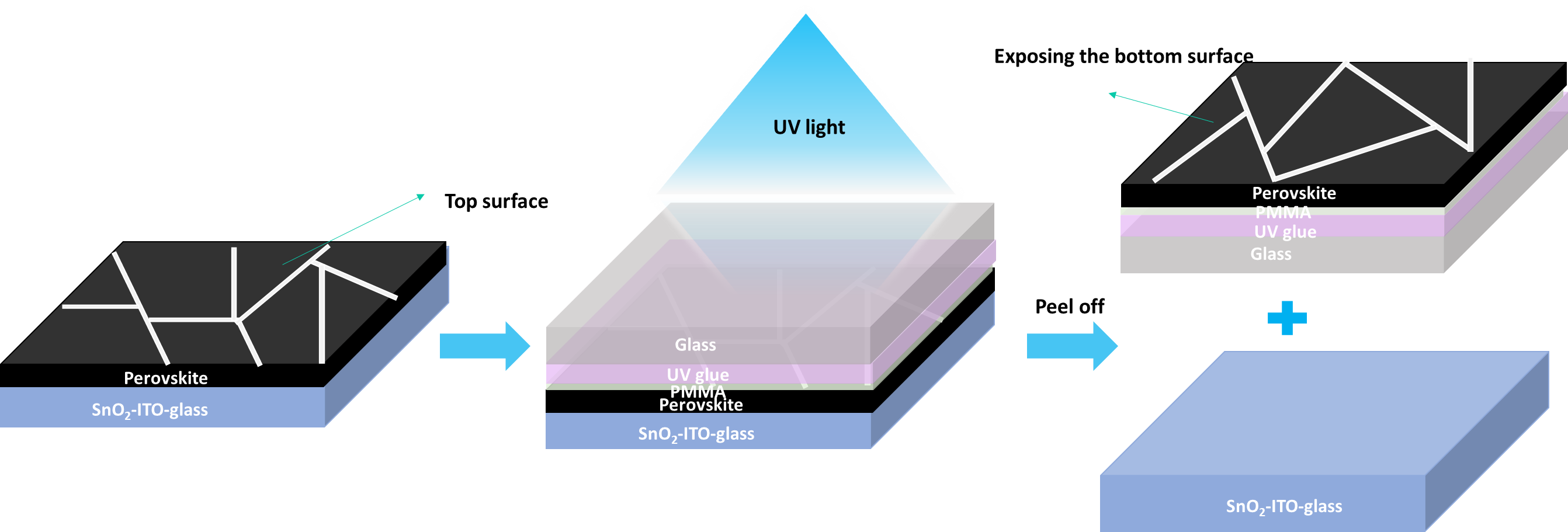


**Fig. S15** The preparation process of the buried perovskite film peeled off from SnO_2_-ITO-glass substrate. (The detailed experimental procedure: After preparing a high-quality perovskite film on the substrate (SnO_2_/ITO/glass), a thin PMMA layer was spin-coated onto the perovskite surface to protect it, followed by the application of UV-curable adhesive and covering with a glass slide. The samples were then exposed to UV light for 3-5 min to cure the adhesive. Finally, due to the stronger adhesion of the UV-cured glue compared to the interfacial bonding at the buried interface, the SnO_2_/ITO/glass slide was peeled off to expose the buried interface)

**Fig. S16** XRD patterns of the control and target perovskite films

**Fig. S17** Magnified XRD patterns with Gaussian fit for the (110) crystal plane of the control and target perovskite films

**Fig. S18** UV-vis absorption spectra of the control and target perovskite films

**Fig. S19** *J*_sc_ values of the corresponding devices versus light intensity on a double-logarithmic scale

**Fig. S20** The residual strain of the corresponding diffraction peaks (2*θ*) of the control and target perovskite films as a function of sin^2^ψ

**Fig. S21** *J-V* curves of the control and PSCs modified with different SA concentrations.

We optimized the SA concentration through *J-V* cures and found that the target PSCs exhibit the best performance at 5 mg mL^-1^, hence fixing this concentration for all experiments. Notably, when the SA concentration exceeded 7 mg mL^-1^, the PCE drops significantly, which are primarily caused by two aspects. One the hand, excessive SA can form a thick SA layer at the buried interface, impeding charge transfer, increasing charge recombination and consequently deteriorating device performance. One the other hand, when the SA concentration increases, the excessive nucleation sites during perovskite growth led to a higher number of smaller grains, significantly degrading the quality of the perovskite film

**Fig. S22** Stabilized *J*_sc_ at maximum power point as a function of time of the control and target perovskite devices


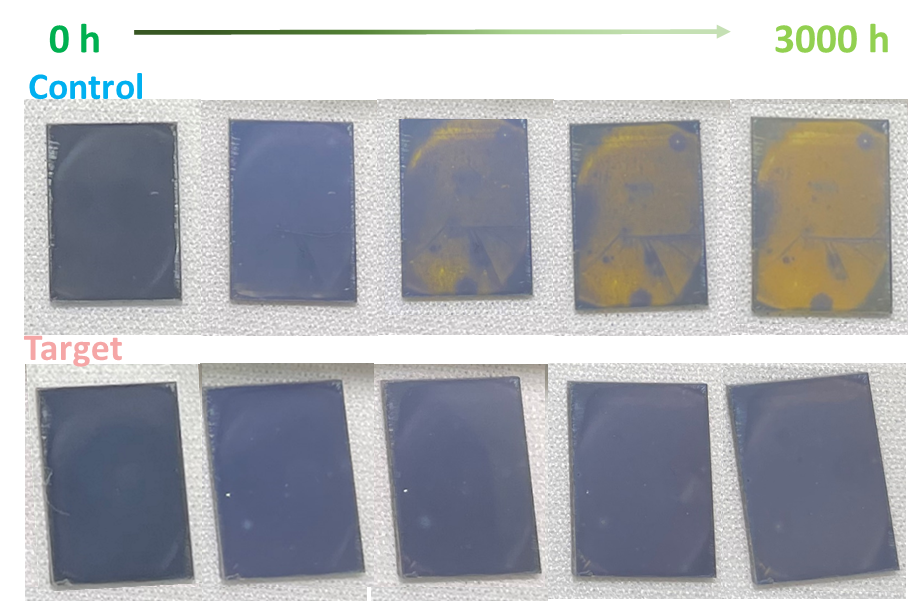


**Fig. S23** Images before and after aging under 45±5% RH of the control and target perovskite films


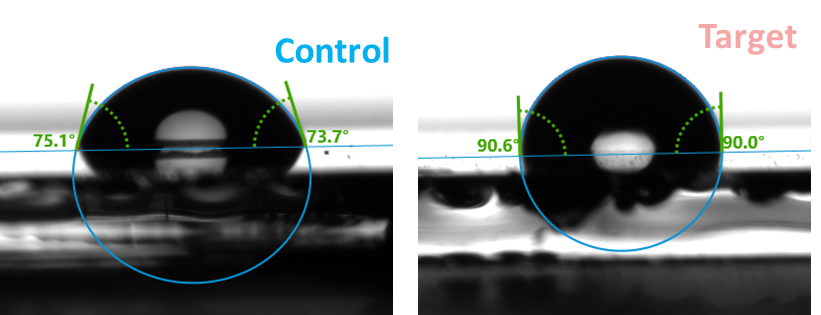


**Fig. S24** Water contact angles of the control and target perovskite films

**Fig. S25** Normalized PCE variation curves of the unencapsulated control and target perovskite devices under 45±5% RH


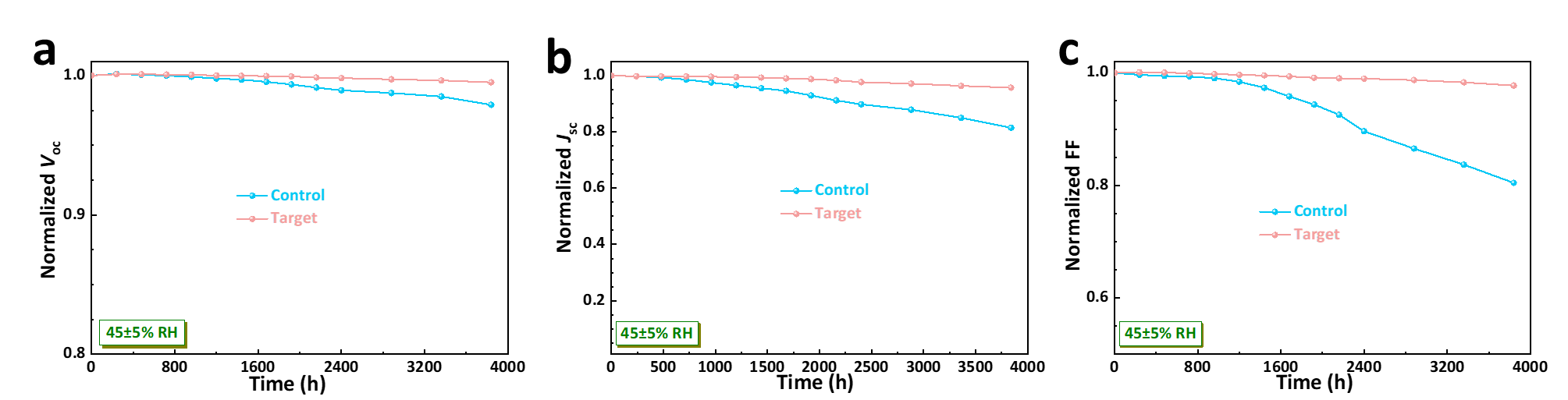


**Fig. S26** Normalized (a) *V*_oc_, (b) *J*_sc_ and (c) FF variation curves of the unencapsulated control and target perovskite devices under 45±5% RH

**Fig. S27** Normalized PCE variation curves of the unencapsulated control and target perovskite devices at 85 °C


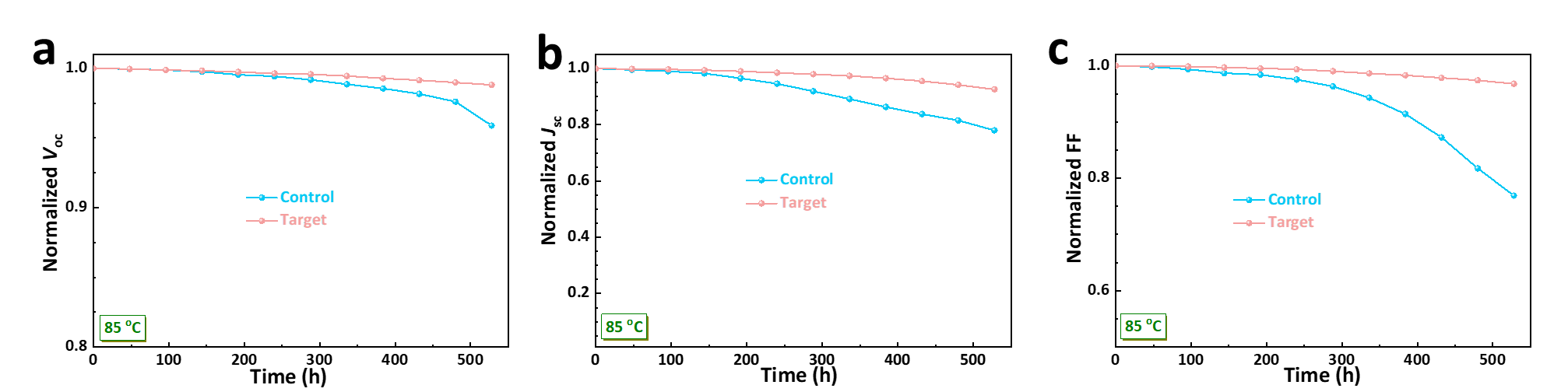


**Fig. S28** Normalized (a) *V*_oc_, (b) *J*_sc_ and (c) FF variation curves of the unencapsulated control and target perovskite devices at 85 °C

**Fig. S29** Normalized absorption intensity variations at 85 °C of the control and target perovskite films


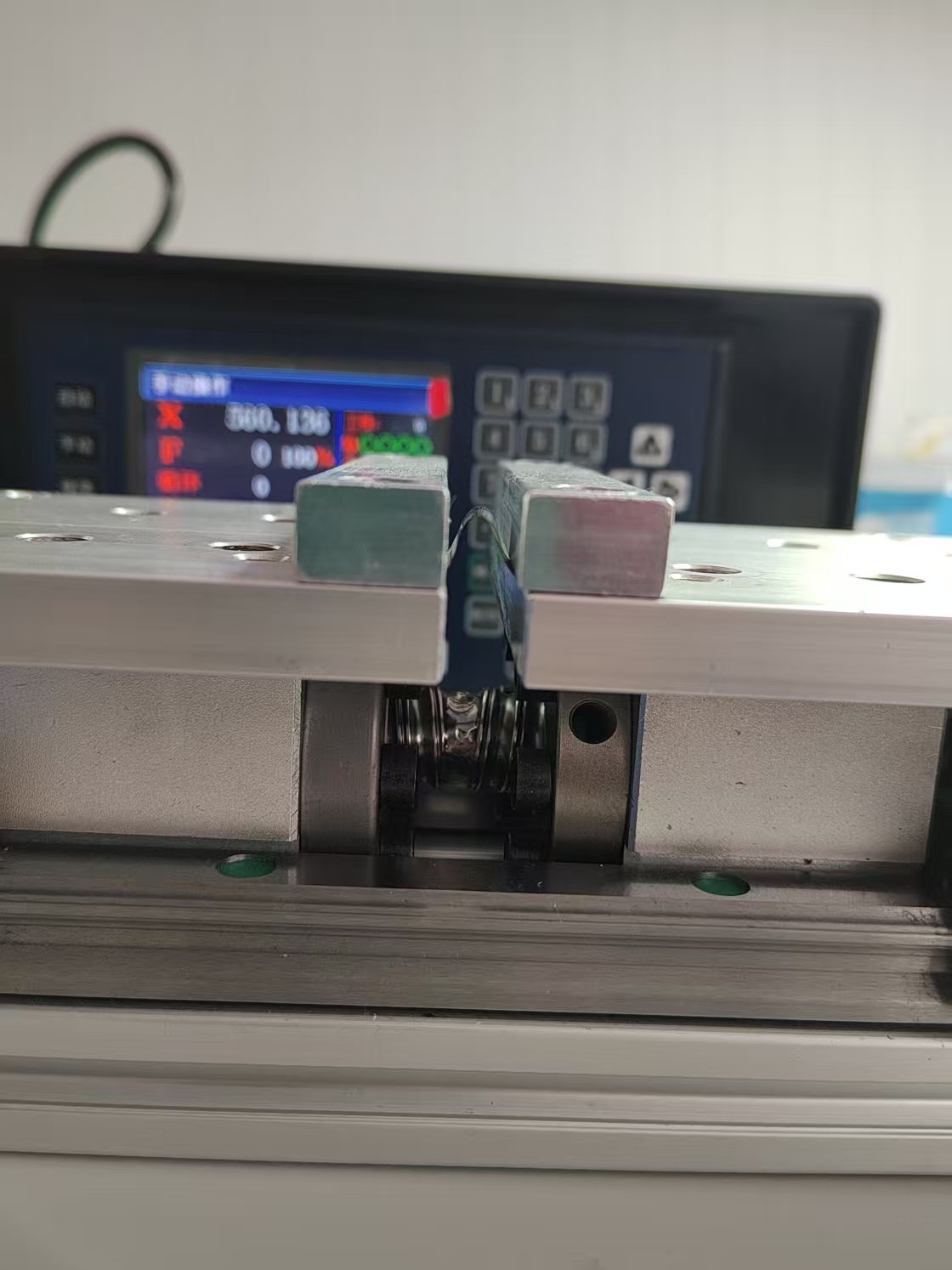


**Fig. S30** Mechanical stability test of flexible perovskite device


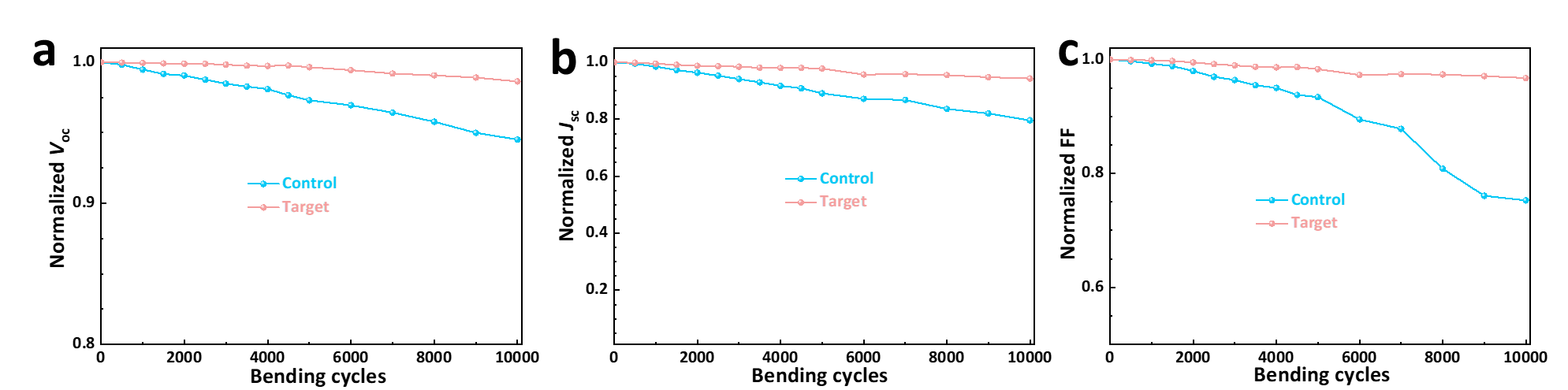


**Fig. S31** Normalized (a) *V*_oc_, (b) *J*_sc_ and (c) FF variation curves of the unencapsulated control and target flexible PSCs over the bending cycle at R= 5 mm

**Table S1** Summary of energies and formation energies (*E*_form_) of various defects calculated using density functional theory

| Structures | Energy (eV) | | | *E*_slab_ (eV) | *E*_atom_ (eV) | | | *E*_form_ (eV) |
| --- | --- | --- | --- | --- | --- | --- | --- | --- |
| FAI-SnO_2_ | V_FA_ | -1224.98 | | -1271.15 | FA | | -42.20 | 3.97  1.60  3.15 |
|  | V_I_ | -1267.96 | |  | I | | -1.59 |  |
|  | V_O_ | -1263.04 | |  | O | | -4.96 |  |
| FAI-SA-SnO_2_ | V_FA_ | -1293.62 | | -1339.94 | FA | -42.20 | | 4.12  2.90  3.34 |
|  | V_I_ | -1335.45 | |  | I | -1.59 | |  |
|  | V_O_ | -1331.64 | |  | O | -4.96 | |  |
| PbI-SnO_2_ | Pb_I_ | | -1306.73 | -1308.26 | Pb | -3.78 | | 3.73 |
|  |  |  |  |  | I | -1.59 | |  |
|  | V_I_ | | -1305.24 |  | I | -1.59 | | 1.44 |
|  | V_Pb_ | | -1300.15 |  | Pb | -3.78 | | 4.33 |
|  | V_O_ | | -1301.22 |  | O | -4.96 | | 2.09 |
| PbI-SA-SnO_2_ | Pb_I_ | | -1339.39 | -1341.21 | Pb | -3.78 | | 4.02 |
|  |  |  |  |  | I | -1.59 | |  |
|  | V_I_ | | -1337.95 |  | I | -1.59 | | 1.67 |
|  | V_Pb_ | | -1332.66 |  | Pb | -3.78 | | 4.77 |
|  | V_O_ | | -1333.98 |  | O | -4.96 | | 2.27 |

**Table S2** Summary of the energy level arrangement for SnO_2_ and SnO_2_+SA

| Films | *E*_cutoff_ (eV) | *E*_onset_ (eV) | *E*_g_ (eV) | *E*_v_ (eV) | *E*_F_ (eV) | *E*_c_ (eV) |
| --- | --- | --- | --- | --- | --- | --- |
| SnO_2_ | 16.82 | 3.86 | 3.99 | 8.24 | 4.38 | 4.25 |
| SnO_2_+SA | 16.99 | 3.88 | 3.93 | 8.09 | 4.21 | 4.16 |

**Table S3** The intensities and FWHM values of the (110) crystal plane for the control and target perovskite films

| Device | Intensity (a.u.) | FWHM |
| --- | --- | --- |
| Control | 7087 | 0.17027 |
| Target | 9775 | 0.14857 |

**Table S4** *R*_ct_ and *R*_rec_ values obtained by fitting the EIS

| PSCs | Control | Target |
| --- | --- | --- |
| *R*_ct_ (Ω) | 892.46 | 516.01 |
| *R*_rec_ (Ω) | 1511.54 | 2323.42 |

**Table S5** Photovoltaic parameters of the control and PSCs modified with different SA concentrations (0.049 cm^2^ aperture) under 1 sun simulated irradiation with a scan speed of 0.05 V*s^-1^

| Concentrations (mg mL^-1^) | *V*_oc_ (V) | *J*_sc_ (mA cm^-2^) | FF (%) | PCE (%) |
| --- | --- | --- | --- | --- |
| Control | 1.17 | 25.04 | 79.46 | 23.19 |
| 3 | 1.17 | 25.29 | 82.00 | 24.35 |
| 5 | 1.19 | 25.47 | 84.30 | 25.50 |
| 7 | 1.18 | 25.41 | 81.17 | 24.29 |
| 10 | 1.16 | 25.16 | 78.72 | 23.00 |

**Table S6** Photovoltaic parameters of the control and target rigid PSCs (0.049 cm^2^) under reverse and forward scan directions with a scan speed of 0.05 V*s^-1^

| Device | *V*_oc_ (V) | *J*_sc_ (mA cm^-2^) | FF (%) | PCE (%) | Hysteresis factor (%) |
| --- | --- | --- | --- | --- | --- |
| Control-Reverse | 1.17 | 25.04 | 79.46 | 23.19 | 3.10 |
| Control-Forward | 1.16 | 24.68 | 78.16 | 22.47 |  |
| Target-Reverse | 1.19 | 25.47 | 84.30 | 25.50 | 0.94 |
| Target-Forward | 1.19 | 25.36 | 83.93 | 25.26 |  |

**Table S7** Photovoltaic parameters of the control and target flexible PSCs (0.049 cm^2^) under reverse and forward scan directions with a scan speed of 0.05 V*s^-1^.

| Device | *V*_oc_ (V) | *J*_sc_ (mA cm^-2^) | FF (%) | PCE (%) | Hysteresis factor (%) |
| --- | --- | --- | --- | --- | --- |
| Control-Reverse | 1.17 | 24.79 | 78.31 | 22.70 | 3.44 |
| Control-Forward | 1.16 | 24.43 | 77.21 | 21.92 |  |
| Target-Reverse | 1.19 | 24.95 | 84.18 | 24.92 | 1.57 |
| Target-Forward | 1.18 | 24.81 | 83.47 | 24.53 |  |

**Table S8** Photovoltaic parameters of the control and target rigid PSCs (1 cm^2^) under reverse and forward scan directions with a scan speed of 0.05 V*s^-1^

| Device | *V*_oc_ (V) | *J*_sc_ (mA cm^-2^) | FF (%) | PCE (%) | Hysteresis factor (%) |
| --- | --- | --- | --- | --- | --- |
| Control-Reverse | 1.16 | 24.52 | 77.74 | 22.07 | 5.57 |
| Control-Forward | 1.15 | 23.84 | 75.80 | 20.84 |  |
| Target-Reverse | 1.18 | 25.11 | 81.11 | 24.01 | 2.46 |
| Target-Forward | 1.17 | 24.83 | 80.28 | 23.42 |  |

**Table S9** Original photovoltaic parameters of the control and target perovskite devices for long-term stability tests

|  | Devices | *V*_oc_ (V) | *J*_sc_ (mA cm^-2^) | FF (%) | PCE (%) |
| --- | --- | --- | --- | --- | --- |
| Humidity stability | Control | 1.17 | 24.96 | 79.23 | 23.07 |
|  | Target | 1.19 | 25.34 | 83.55 | 25.24 |
| Thermal stability | Control | 1.17 | 24.87 | 79.25 | 23.03 |
|  | Target | 1.19 | 25.42 | 83.12 | 25.14 |
| MPPT measurement | Control | 1.16 | 25.07 | 78.99 | 22.98 |
|  | Target | 1.19 | 25.14 | 84.55 | 25.22 |
| Bending stability | Control | 1.16 | 24.23 | 76.81 | 21.58 |
|  | Target | 1.18 | 24.69 | 83.68 | 24.38 |
